# Editorial: Proprioception in sports and health

**DOI:** 10.3389/fnhum.2025.1646186

**Published:** 2025-07-01

**Authors:** Daniel Rojas-Valverde, Pantelis T. Nikolaidis, Andrea Fallas-Campos

**Affiliations:** ^1^Centro de Investigación en Salud y Deporte (CIDISAD), Escuela Ciencias del Movimiento Humano y Calidad de Vida (CIEMHCAVI), Universidad Nacional, Heredia, Costa Rica; ^2^Clínica de Lesiones Deportivas (Rehab&Readapt), Escuela Ciencias del Movimiento Humano y Calidad de Vida (CIEMHCAVI), Universidad Nacional, Heredia, Costa Rica; ^3^School of Health and Welfare Sciences, University of West Attica, Athens, Greece; ^4^Facultad Ciencias del Deporte, Universidad de Extremadura, Cáceres, Spain

**Keywords:** motor control, exercise therapy, postural balance, rehabilitation, sports performance, sports injuries

## Introduction

Proprioception is the body's ability to perceive its position and movement in space and time (Munóz-Jiménez et al., 2021; Yilmaz et al., 2024); it is a cornerstone of motor control, postural regulation, and injury prevention. This Research Topic highlights the pivotal role of proprioception not only in athletic performance but also in broader domains of health, rehabilitation, and injury prevention. As individuals age, the proprioceptive function becomes increasingly critical for maintaining balance, posture, mobility, reducing the risk of falls, and preserving autonomy; factors that directly influence the quality of life (Aleixo and Abrantes, 2024; Ferlinc et al., 2019). In sports, proprioceptive exercises should be integrated both within and beyond regular training sessions to help athletes enhance body awareness, reduce injury risk, and optimize power transfer (Yilmaz et al., 2024). This Research Topic compiles and analyses five key articles that explore the diverse applications of proprioception. The Research Topic includes three original research articles, one opinion article, and one review, offering a multifaceted perspective on proprioceptive mechanisms across clinical and athletic contexts.

One of the included studies systematically reviewed the effects of proprioceptive exercise in individuals with knee osteoarthritis (Lin et al.). While proprioceptive training is widely employed in the management of knee osteoarthritis, its efficacy has remained a topic of debate. This study aimed to systematically assess the impact of proprioceptive exercise on symptom relief and functional outcomes in individuals with knee osteoarthritis. The study found that proprioceptive interventions significantly improved overall balance function and certain pain assessments, particularly in the short term. These findings suggest that proprioceptive exercise offers meaningful yet selective benefits for patients with knee osteoarthritis. This finding aligns with previous studies that support the notion that exercise stimulates proprioceptors located in muscles, tendons, and joints, thereby enhancing the body's ability to detect position (Heroux et al., 2022; Yilmaz et al., 2024). This heightened proprioceptive feedback is critical for adjusting posture and maintaining stability during both static and dynamic activities.

In this Research Topic, proprioception was examined through the lens of kinesiology taping, an increasingly popular method in musculoskeletal rehabilitation (Zhou et al.). The study evaluated the effect of kinesiology taping on balance, specifically when applied to the quadriceps. While static balance remained unaffected, dynamic balance saw significant improvement with taping. This outcome suggests that proprioceptive cues delivered through kinesiology taping may enhance neuromuscular readiness and reactive control, particularly in tasks requiring rapid postural adjustments.

To further understand proprioceptive modulation, another study examined high-definition transcranial direct current stimulation (HD-tDCS) over the parietal lobes and its effect on postural control (Yang et al.). Here, proprioception is explored from a central nervous system standpoint, emphasising cortical processing rather than peripheral feedback. Results indicated that HD-tDCS, especially bilateral stimulation, significantly influenced postural latency and motor response under different sensory conditions. These findings reinforce the concept that proprioception is not only mechanical but also neurocognitive, modulated by both somatosensory and visual inputs processed at cortical levels. This reinforces the notion that high-level proprioceptive judgments exist, diverging from the traditional understanding of proprioception; its conceptualisation, physiological cascade, and network (Heroux et al., 2022). It also challenges the conventional methods used to assess proprioceptive abilities, particularly those related to higher-order processing (Munóz-Jiménez et al., 2021).

Shifting to a more unconventional setting, another case study explored proprioceptive dolphin-assisted activities as a therapeutic modality for adults with major depressive disorder (Kreiviniene et al.). The two-week intervention led to improvements in physical function and specific dimensions of quality of life, accompanied by measurable changes in muscle tone and relaxation. Notably, proprioceptive input through multisensory aquatic environments appeared to elicit both physical and emotional adaptations. This study highlights the psychosomatic potential of proprioception in modulating stress, mood, and neuromuscular responses. Another indication is that brain functions, particularly those related to proprioception, are increasingly understood as centrally mediated abilities rather than purely peripheral ones. The evidence suggests that emotional states have a distinct impact on proprioceptive accuracy and postural stability, providing new insights into the complex interplay between emotions and motor control (Riquelme et al., 2024; Yuan et al., 2025).

Finally, proprioception also plays a critical role in managing acute and chronic sports injuries. As we move from joint-specific disorders to broader athletic concerns, a novel perspective article introduced the interplay of the concepts of reversible involution and cacostasis, a stress-induced physiological regression state, in the context of sports injuries (Rojas-Valverde et al.). The integration of proprioceptive training within this framework supports its role not only in structural recovery but also in facilitating neurocognitive and emotional rebalancing. This positions proprioception as a mediator between physical rehabilitation and systemic adaptation, contributing to a more holistic approach to athlete care. Cacostasis can lead to the dysregulation of neuromuscular feedback mechanisms, which are essential for proprioception. This loss of precision and synchronisation in the perception of movement and joint position increases the risk of erratic or unanticipated movements, which can lead to injuries such as sprains, tears, and dislocations (Bornstein et al., 2021). Furthermore, cacostasis reduces this adaptive capacity of the nervous system, hindering effective rehabilitation of proprioception after an injury and increasing the risk of relapse or compensatory injuries. According to the authors, this could lead to a state of sensorimotor involution, which must be addressed as a crucial part of the rehabilitation process.

**Figure 1:**
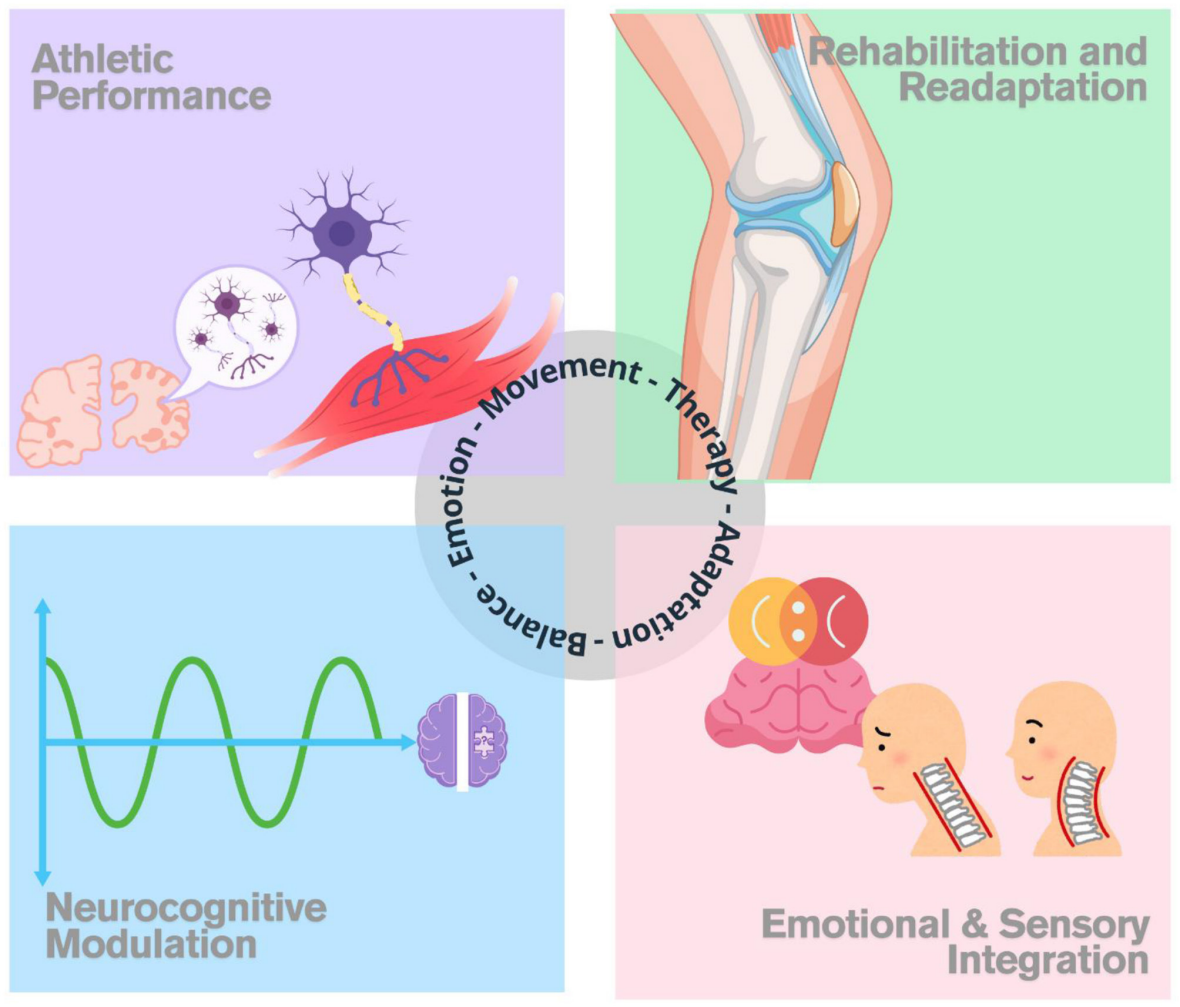
Integrated functional domains of proprioception. This figure illustrates four key domains where proprioception plays a central role: (1) athletic performance through neuromuscular coordination; (2) rehabilitation and readaptation processes following injury; (3) neurocognitive modulation related to cortical patterns and sensorimotor perception; and (4) emotional and sensory integration linking affective states with postural and movement control. These domains converge along a therapeutic axis grounded in emotion, movement, adaptation, and balance.

In conclusion, proprioception emerges as a multidimensional construct with implications that extend far beyond traditional motor control paradigms. The studies compiled in this Research Topic underscore its relevance across clinical, athletic, neurological, and even psychosocial domains (see Figure 1). Collectively, these five studies demonstrate the breadth and versatility of proprioception in health and sports sciences. From degenerative joint conditions and elite athletic rehabilitation to neurostimulation and mental health interventions, proprioception emerges as a critical and adaptable component of human function. The research advances presented in this topic not only expand our understanding of proprioceptive mechanisms but also encourage innovative strategies for training, therapy, and performance enhancement across diverse populations.
